# Mechanics of Channel Gating of the Nicotinic Acetylcholine Receptor

**DOI:** 10.1371/journal.pcbi.0040019

**Published:** 2008-01-25

**Authors:** Xinli Liu, Yechun Xu, Honglin Li, Xicheng Wang, Hualiang Jiang, Francisco J Barrantes

**Affiliations:** 1 Department of Engineering Mechanics, State Key Laboratory of Structural Analysis for Industrial Equipment, Dalian University of Technology, Dalian, Liaoning, China; 2 Drug Discovery and Design Center, State Key Laboratory of Drug Research, Shanghai Institute of Materia Medica, Chinese Academy of Sciences, Shanghai, China; 3 School of Pharmacy, East China University of Science and Technology, Shanghai, China; 4 UNESCO Chair of Biophysics & Molecular Neurobiology and Instituto de Investigaciones Bioquímicas de Bahía Blanca, Bahía Blanca, Argentina; University of Houston, United States of America

## Abstract

The nicotinic acetylcholine receptor (nAChR) is a key molecule involved in the propagation of signals in the central nervous system and peripheral synapses. Although numerous computational and experimental studies have been performed on this receptor, the structural dynamics of the receptor underlying the gating mechanism is still unclear. To address the mechanical fundamentals of nAChR gating, both conventional molecular dynamics (CMD) and steered rotation molecular dynamics (SRMD) simulations have been conducted on the cryo-electron microscopy (cryo-EM) structure of nAChR embedded in a dipalmitoylphosphatidylcholine (DPPC) bilayer and water molecules. A 30-ns CMD simulation revealed a collective motion amongst C-loops, M1, and M2 helices. The inward movement of C-loops accompanying the shrinking of acetylcholine (ACh) binding pockets induced an inward and upward motion of the outer β-sheet composed of β9 and β10 strands, which in turn causes M1 and M2 to undergo anticlockwise motions around the pore axis. Rotational motion of the entire receptor around the pore axis and twisting motions among extracellular (EC), transmembrane (TM), and intracellular MA domains were also detected by the CMD simulation. Moreover, M2 helices undergo a local twisting motion synthesized by their bending vibration and rotation. The hinge of either twisting motion or bending vibration is located at the middle of M2, possibly the gate of the receptor. A complementary twisting-to-open motion throughout the receptor was detected by a normal mode analysis (NMA). To mimic the pulsive action of ACh binding, nonequilibrium MD simulations were performed by using the SRMD method developed in one of our laboratories. The result confirmed all the motions derived from the CMD simulation and NMA. In addition, the SRMD simulation indicated that the channel may undergo an open-close (O ↔ C) motion. The present MD simulations explore the structural dynamics of the receptor under its gating process and provide a new insight into the gating mechanism of nAChR at the atomic level.

## Author Summary

Nicotinic acetylcholine receptors (nAChRs) play an essential role in the propagation of signals in the central nervous system and peripheral synapses. However, their gating mechanism is still not fully understood. The cryo-EM structure of nAChRs provides a clue, but the dynamics of receptor structure remains to be elucidated. Using conventional molecular dynamics (CMD), normal mode analysis, and nonequilibrium MD techniques, we investigated the mechanical fundamentals of nAChR gating. Through the simulations, we detected the collective motions of the ligand-binding sites, extracellular (EC) domain, transmembrane (TM) domain, and intracellular (MA) domain, which together synthesize a local twisting motion of the M2 helices. To mimic the pulsive action of ligand binding, rotational motion of the receptor was simulated with the steered rotation MD (SRMD), a nonequilibrium MD method developed by us. The results indicate that the channel may undergo an open–close motion along with the rotation of the EC domain. Our study provides a clear picture of the correlation between the structural dynamics of nAChR and its gating mechanism.

## Introduction

The nicotinic acetylcholine receptor (nAChR) is a key molecule involved in the propagation of signals between nerve cells and their targets at the central nervous system and peripheral synapses. As a paradigm of the ligand-gated ion channels (LGICs) superfamily, nAChR is a member of the Cys-loop family which includes the 5-hydroxytryptamine type 3 (5-HT_3_) receptor, γ-aminobutyric acid type A (GABA_A_) and GABA_C_ receptors, and glycine (inhibitory) receptors [1]. nAChRs have been identified as crucial elements in central nervous system functions such as consciousness, attention, and memory; and participate in numerous cerebral circuits. Alteration of these functions gives rise to various invalidating pathologies such as Alzheimer disease [2], Parkinson disease [3], and schizophrenia [4]. nAChRs are therefore increasingly becoming very important drug targets [2–10].

The nAChR has a special place in the history of ion channels since it has scored a number of “firsts”: it was the first ion channel to be purified, to be sequenced, to be functionally reconstituted in synthetic lipid bilayers, to be cloned, and to be recorded the electrical signal as a single open channel [11]. Furthermore, it is the only ligand-gated channel to have an experimentally determined three-dimensional structure [12]. This physiologically and pharmacologically important receptor has been used as a model for studying the structure–function relationship of the ligand-gated ion channels [1,5,6,12].

Structurally, nAChR has four homologous subunits with a stoichiometry α_2_βγδ in its embryonic form and α_2_βɛδ in the adult, assembled pseudosymmetrically as three concentric rings around a central ion-permeation channel [13]. The nAChR is composed of three domains: a large N-terminal extracellular (EC) domain, a transmembrane (TM) domain, and a smaller intracellular domain [12], forming an architecture of ∼160 Å in length in the direction perpendicular to the membrane plane. The acetylcholine (ACh) binding sites lie at the subunit interfaces in the EC domain, and the M2 helices constitute an axial cation-specific channel in the TM domain [14]. The innermost ring of five M2 segments forms the wall of the nAChR ion channel pore.

Before the structure of the nAChR from Torpedo marmorata was determined, the crystal structure of the acetylcholine binding protein (AChBP) from Lymnaea stagnalis [15] and the electron microscopy (EM) structure of the *Torpedo* nAChR TM domain [16] were used as structural models for studying the structure–function relationship of nAChR. Indeed, several simulations have been performed on these structural models [17]. A 35-ns conventional molecular dynamics (CMD) simulation has been performed to investigate the conformational dynamics of the entire TM domain of the receptor [18]. A blocking mechanism of the noncompetitive inhibitor of chlorpromazine to the ion channel pore of the nAChR has been explored at the atomic level using both conventional and steered MD simulations [19]. Additionally, both CMD and nonequilibrium MD simulations have been performed on the modeling structure of the neuronal-type nAChR with five α7 subunits [20]. More recently, the refined cryo-EM structure of nAChR at 4 Å resolution has provided additional information on the whole picture of the receptor [12].

One of the remaining challenges in this area is to fully elucidate the gating mechanism of the nAChR, which is associated with almost all of its functions [21]. Although the moderate resolution cryo-EM structure of nAChR provides a picture of receptor structure [12], there are still some gaps in our knowledge. For example, some results obtained with substituted cysteine or histidine accessibility methods suggested that the position of the gate is near the cytoplasmic limit of M2 [22–24], whereas other experimental results revealed the gate to be in the middle of M2 [25,26]. In particular, the dynamics of receptor structure underlying the gating mechanism is still unclear.

Understanding the structural dynamics of nAChR at the atomic level is of great physiological and pharmacological relevance. Investigating such a large protein assembly embedded in the lipid bilayer using MD simulation poses enormous challenges, and in many cases these dynamic conformational changes are beyond the scope of the CMD methods. For this reason, in the present study the dynamic behavior of nAChR has been studied using CMD in conjunction with normal mode analysis and a special nonequilibrium MD method developed by our group. Taking into account the special dynamic motion of nAChR spanning the lipid bilayer, we developed a nonequilibrium MD method, named Steered Rotation Molecular Dynamics (SRMD), to simulate the rotation of nAChR around the principal axis along the channel. In the following, we report on the MD results for the dynamic motions of nAChR in correlation with its gating mechanism.

## Results

### Dynamics of nAChR Revealed by CMD Simulation

**Figure 1:**
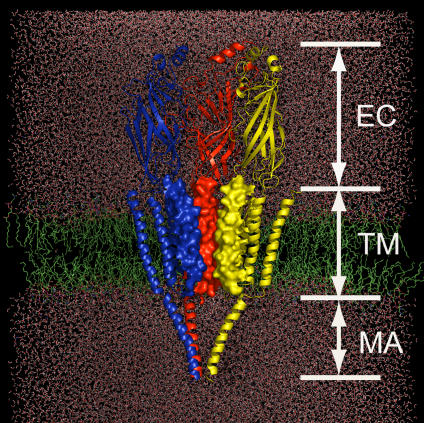
Schematic Representation of the Simulation System Constructed on the Basis of the Cryo-EM Structure of nAChR at 4 Å Resolution nAChR is embedded in a DPPC lipid bilayer (green) and water molecules (red). Only three subunits (color-coded in red, blue, and yellow) are displayed for clarity. The M2 helices are rendered with electrostatic potential surface to highlight the pore shape.

The nAChR was embedded in a dipalmitoylphosphatidylcholine (DPPC) bilayer [18,27], the nAChR/DPPC complex was solvated using a bath of the simple point charge (SPC) water molecules [28], and the whole system was neutralized by adding numbers of Na^+^ and Cl^−^ ions, giving rise to a final simulation system containing more than 250,000 atoms. The schematic representation of the simulation system is shown in Figure 1. Firstly, the system was equilibrated by running a short (950 ps) MD simulation. The root mean square deviation (RMSD) between the final equilibrated structure of nAChR and the original structure is 1.02 Å. A 30-ns CMD simulation was subsequently performed and the MD trajectory was used to analyze the conformational dynamics of the receptor.

**Figure 2:**
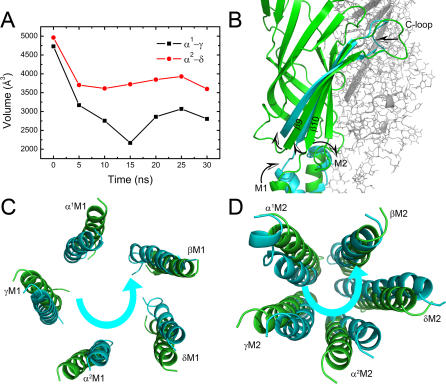
Collective Motions of the C-Loops, M1, and M2 Helices (A) The time-dependence of the volumes of ACh binding pockets. Black and red curves represent type-I (α^1^-γ) and type-II (α^2^-δ) pockets, respectively. (B) The motion tendencies of C-loops, M1, and M2 reflected by the superposition of subunit α^1^ at the beginning (green) and after 30 ns simulation (cyan). The picture is displayed parallel to the pore axis. (C–D) The anticlockwise motions of the M1 and M2 helices. The superpositions are based on the centers of mass of the five M1 and M2 helices, respectively, and the pictures are viewed from the synaptic cleft.

ACh binding is essential to the gating of nAChR, which induces a conformational change that opens the channel [16,29]. We therefore first of all investigated the dynamics of the ACh binding pockets. For the subtype of nAChR we studied, there are two binding pockets located at the interfaces between α^1^ and γ subunits (type-I) and between α^2^ and δ subunits (type-II), respectively [12,30]. These two binding pockets were monitored throughout the 30-ns CMD simulation by using the SURFNET program [31]. As indicated in Figure 2A, both type-I and type-II ACh binding pockets shrunk significantly during the 30-ns CMD simulation. The α-subunit C-loops act as lids of the binding pockets. The flexibility of C-loops was observed experimentally [32] and theoretically [20,33]. Our CMD simulation indicates that the C-loop of each α-subunit moves inward toward the center of the molecule (Figure 2B); this motion is the major cause of shrinkage of the ACh binding pocket. Concomitantly, the inward motion of C-loops drives the outer β-sheet (made up of strands β9 and β10) inward and upward, in particular the bottom part, as shown in Figure 2B. This motion further stimulates the movement of M1 (Figure 2B). Taking the movements of the five subunits together, it can be seen that the five M1 helices perform an anticlockwise rotation around the pore axis, as viewed from the top of the receptor (Figure 2C). Since M2 connects directly with M1 through a loop, the rotation of M1 should induce M2 to rotate around the pore axis in the same direction. Indeed, viewed from the synaptic cleft, the 5-fold M2 helices rotate in an anticlockwise manner around the pore axis, as shown in Figure 2D. Accordingly, the 30-ns CMD simulation detected a clear correlation between the motions of the ACh binding pockets, C-loops, and the M1 and M2 helices.

**Figure 3:**
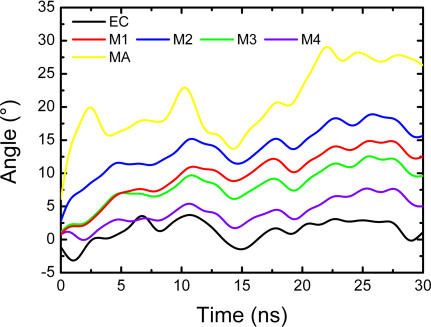
The Time-Dependence of the Rotational Angles of the EC domain, M1–M4 and MA helices, with Respect to Their Starting Structures

Taking into account the rotating motions of M1 and M2 helices, we subsequently investigated the circumgyrations of the other components of nAChR by determining the rotation angles of the EC domain, TM, and intracellular MA helices. The rotation angle of each body as a whole (for instance the five M1 or M2 helices) was derived rigorously from the moment of inertia. Details of the method used to calculate the rotation angles are given in the Materials and Methods section. As indicated in Figure 3, both TM helices (M1–M4) and non-TM MA helices undergo global anticlockwise rotations with respect to the EC domain. The intracellular MA helices, exposed to the water milieu, experienced the most pronounced rotation of all nAChR helices, whereas the EC domain is the most static part of the whole receptor. The rotation of the M2 helices in the TM domain is more pronounced than that of other TM segments, which is in agreement with previous simulation result [34] and supports the viewpoint that the innermost M2 ring constitutes a distinct assembly within the TM region [35]. The second most pronounced rotation among TM segments corresponds to the M1 helices. The M3 and M4 helices rotate relatively slower in comparison with M1 and M2 helices.

**Figure 4:**
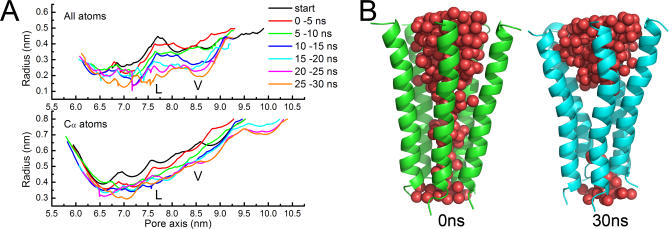
Changes of the Pore Channel (A) Pore radius profiles versus position along the pore axis. Curves represent the average radii changes over different time intervals for the all-atoms (top) and C_α_ atoms (bottom) of the five M2 helices. (B) The volume change of the pore channel reflected by the water molecules embedded within the channel.

M2 helices line the innermost wall of the channel and thus their motions and conformational changes are directly associated with the gating of the receptor. Changes in channel radii along the pore were monitored during the 30-ns CMD trajectory using the HOLE program [36–38]. The results indicate that the channel undergoes a visible reduction, as reflected by the changes in the radius profiles (Figure 4A). Consistent with the conclusion from earlier, lower resolution cryo-EM structure of the nAChR [16], the extent of decreases in channel radii is most prominent in the conserved hydrophobic regions around the L251 and V255 rings (L- and V-rings). Furthermore, the radius change profiles indicate that the side-chains of inner residues of M2 helices move more deeply toward the pore axis than do the C_α_ atoms (Figure 4A). The shrinkage of the pore channel is also reflected by the change in the amount of water molecules inside the channel. As shown in Figure 4B, the number of intrachannel water molecules decreased from 181 to 134 after 30 ns simulation. Particularly remarkable was the narrowing of the middle segment of M2 helices around the L- and V-rings, turning into *vacuo*.

**Figure 5:**
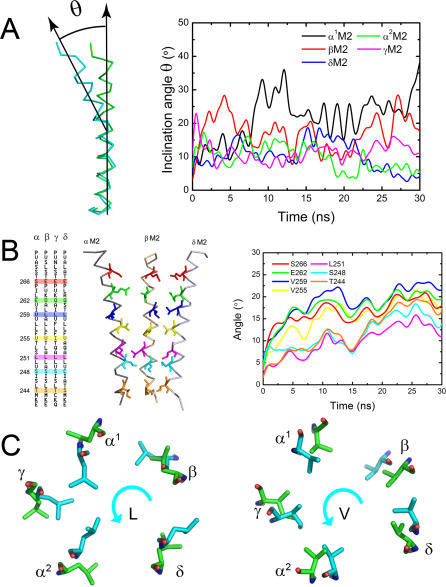
Dynamics of the M2 Helices (A) Bending vibrations of the M2 helices represented by changes of inclination angle along the CMD trajectory. (B) Schematic representation for the positions of some important residues throughout the M2 helices (left). Rotation angle profiles of the important residues (right). (C) Rotations of the L- and V-rings. The anticlockwise rotations of L- and V-rings are represented by the conformational superposition between the starting structure (green) and final structure of the CMD simulation (cyan) on the receptor.

Figure 4B indicates that M2 helices possibly engage in a bending motion in addition to rotation. Indeed, inspection of the conformational changes in M2 helices along the CMD trajectory revealed a significant bending motion. Although previous studies have also detected the bending motion of M2 helices [34], our 30-ns CMD simulation provides more detailed information. For each M2 helix, the bending hinge is located between L251 and V255. Thus, the pore channel is divided into two parts by the hinge. We describe the bending of each M2 helix by the inclination angle of the top part of the helix with respect to the bottom part, as illustrated in Figure 5A. The inclination angle profiles indicate that each M2 helix undergoes a bending vibration, and the amplitude of α^1^M2 helix is the largest (Figure 5A). A recent single-molecule kinetic analysis on αM2 of nAChR suggested that most of the residues throughout the length of αM2 move relatively early in the gating reaction, but some at the mid-section move later [26]. This experimental result implies that the mid-section moves slower than the other part does, i.e., the αM2 might bend round the hinge during the gating process. This result gave an indirect support to the bending vibration of M2 helices, especially α^1^M2, as detected by our CMD simulation.

Intuitively, the combination of bending and rotational motions should produce a twisting motion. We therefore re-inspected the rotation of M2 helices by determining the rotation angles of some typical residues along the helices. The results are shown in Figure 5B, and the positions of the residues throughout the helices are labeled in the left of Figure 5B. Figure 5C shows the rotational motions of the residues composing the L- and V-rings. Interestingly, divided by the hinge, the top part of each M2 rotates faster than the bottom part, resulting in a twisting motion of the M2 helices around the hinge. This implies that location around the hinge is important for controlling the gating of the receptor. Furthermore, this simulation result agrees with the recent single-molecule kinetic analysis study on the αM2 of nAChR, indicating that a gate is located at the middle of M2 [26]. Thus, the bending and rotational motions of M2 helices as well as their synthetic twisting motion provide a structural and dynamic basis for channel constriction and dilation, which may be associated with the gating mechanism as will be discussed below.

### Dynamics of nAChR Revealed by Normal Mode Analysis (NMA)

**Figure 6:**
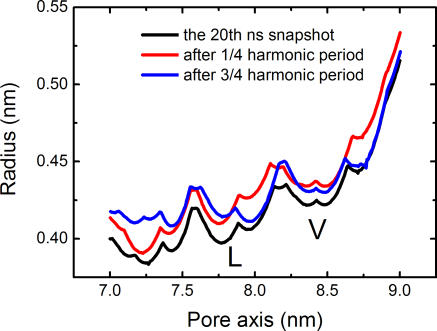
Pore Radius Profiles versus Position along the Pore Axis Three curves were produced on the basis of C_α_ atoms of M2 helices from the 20th ns snapshot as well as the structures resulting from the NMA on this snapshot.

Recent NMA simulation studies based on a tentative model of the homomeric α7 neuronal nAChR [33,39] and the heteropentameric *Torpedo* nAChR [40] proposed a twist-to-open mechanism for nAChR dynamics. NMA was also applied to the nAChR in the present study to gain insight into the gating mechanism of this receptor. The snapshot at 20 ns on the CMD trajectory was extracted as the structure model for NMA, because the receptor reaches a more closed and stable state after such a lengthy CMD simulation (Figures 4, 5, and S1). The NMA results for the lowest-frequency modes are very similar to those of previous studies on the modeling structures of nAChR [33,39,40]; the detailed description for NMA is shown in the Figures S2–S4. In general, a global twisting motion occurs between the EC domain and the TM domain of nAChR. The first mode yielded symmetrical deformations of the entire nAChR structure. The five subunits of the EC domain and the corresponding M2 helices undergo concerted global rotations in opposite directions around the pore axis (see Figure S2). Other TM segments and MA helices undergo similar periodic rotations (Figure S3). Thus, the quintuple deformation of nAChR can be expressed in a twisting movement involving rotations of the EC and TM domains in opposite directions. Moreover, the global twisting motion of nAChR tends to increase the width of the entire pore, including the L- and V- rings, as indicated in Figure 6. These results are also in agreement with experimental [12,16,41–43] and recent theoretical studies [33,39,40].

### Dynamics of nAChR Revealed by Nonequilibrium MD Simulation

Both CMD and NMA address the twisting motion between the EC and TM domains (Figures 5 and S2). These two kinds of simulations produced opposite changes in pore channel structure: CMD detected a shrinking motion and NMA recognized a tendency for the pore to be open (Figures 4 and 6). Because the NMA was carried out on the basis of the 20th snapshot on the CMD trajectory, i.e., when the nAChR is in a more closed state, the above two pore channel motions are likely to be complementary. This implies that the nAChR channel may undergo an “open–close (O ↔ C)” movement on a physiological time scale. However, it is very difficult for CMD to detect the open–close type motions of nAChR owing to the inaccessibly long time scale the simulation would require, which would be at least as long as the physiological timescale. As mentioned above, the pore channel shrinkage and opening correlate with receptor rotation. Accordingly, to testify the occurrence of the O ↔ C motion of the pore channel, we used a nonequilibrium MD method to simulate the receptor by speeding up the rotation process.

**Figure 7:**
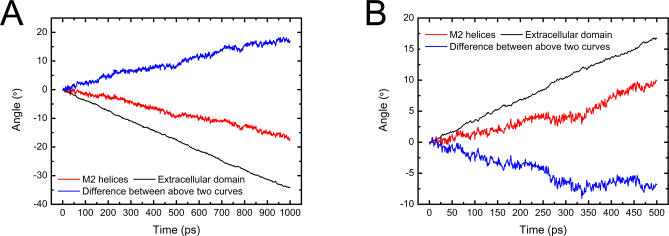
The Time-Dependence of the Rotation Angles of EC Domain and M2 Helices as the Receptor Rotates in Clockwise (A) and Anticlockwise (B) Directions The difference between the rotation angles of EC domain and M2 helices is also shown (blue solid line).

**Figure 8:**
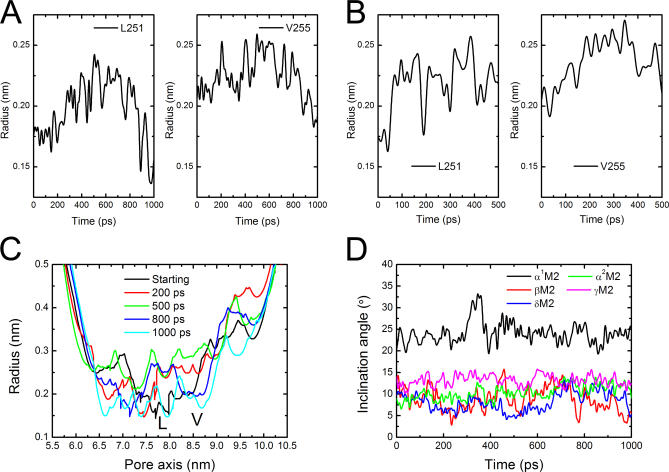
Dynamics of the Pore Channel Derived from the SRMD Simulations (A–B) The time-dependence of the radii of L- and V-rings as the receptor rotates in clockwise and anticlockwise directions, respectively. (C) Pore radius profiles versus position along the pore axis. Curves represent the radius changes at different time intervals for the all-atoms as the receptor rotates in clockwise direction. (D) Bending vibrations of the M2 helices represented by changes of inclination angle along the SRMD trajectory of nAChR rotating in clockwise direction. The inclination angle has been defined in Figure 2A.

**Figure 9:**
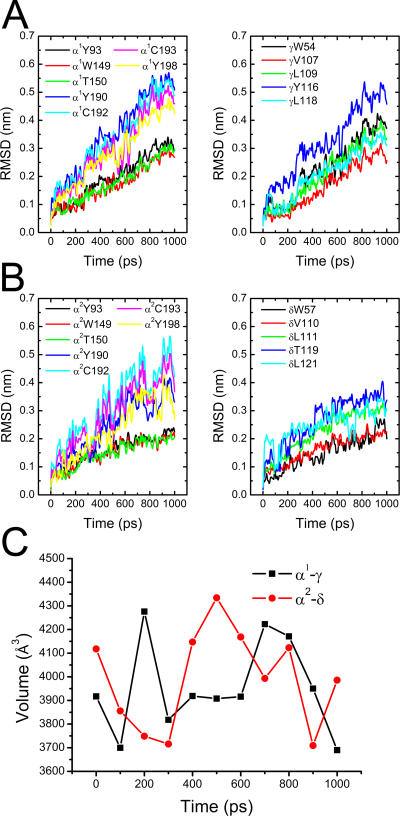
Dynamics of the ACh Binding Pockets Revealed by the SRMD Simulation (A–B) The time-dependence of RMSD values of the conserved residues consisting of type-I and type-II binding pockets, respectively. (C) The time-dependence of volumes of the binding pockets. Black and red curves represent type-I (α^1^-γ) and type-II (α^2^-δ) pockets, respectively.

Existing nonequilibrium MD methods have not thus far been able to simulate the rotation process of the protein, thereby we developed a special nonequilibrium MD method called SRMD (Steered Rotation Molecular Dynamics). The detail of the methodology is given in the following Materials and Methods section. Briefly, during the MD simulation, an angular momentum conserved along the pore axis was assigned to the entire EC domain, enforcing the receptor to rotate around the pore axis. The angular velocity of 5 × 10^−4^ rad·ps^−1^ was imposed on the EC domain to spin the receptor around the pore axis. Two SRMD simulations were then performed: a 1-ns simulation for the clockwise rotation, and a 500-ps simulation for anticlockwise rotation. The results are shown in Figures 7, 8, and 9.

The time-dependences of the rotation angles for the EC domain and M2 helices were monitored during the SRMD simulations (Figure 7). The results clearly demonstrate that the rotation indeed may be handed on from the EC domain to the TM domain. Either the clockwise or the anticlockwise simulation revealed that the TM domain rotates around the pore axis more slowly than the EC domain, producing a twisting motion between the two domains. The radii of L- and V-rings were used to probe pore channel changes following the rotation of the receptor (Figure 8A and 8B). The clockwise rotation produces a clear picture of fluctuation for the L- and V-rings: these two rings distend first and reach peaks at ∼500 ps, gradually shrinking thereafter (Figure 8A). Moreover, the radius profiles along the pore axis at different times of SRMD simulation for clockwise rotation also display a visible open-close (O ↔ C) process of the pore channel (Figure 8C). The displacements of the channel diameters along the SRMD simulation are obvious; the change of pore diameter in the vicinity of the L- and V-rings could be as large as ∼3.0 Å. Although the anticlockwise rotation also detected an O ↔ C motion for the pore channel (Figure 8B), the L-ring changes are out of the step of V-rings, implying that such mode of motion might not actually occur physiologically since it does not match the gating process of the receptor. In addition, the driving force for the receptor rotation is from the binding of ACh to the receptor, and the orientations of the entrances of ACh binding pockets determine that two ACh molecules can only bind to nAChR in a clockwise direction. Thus, it can be concluded that the clockwise rotation reflects the true motion of the receptor under physiological condition. Having obtained the correct rotation orientation, we monitored the motions of TM segments. As with the motions derived from the CMD simulation (Figure 5A), bending vibrations of the M2 helices were also detected by the SRMD simulation (Figure 8D). The amplitudes of these vibrations are smaller than those produced by CMD simulation. Again, α^1^M2 bends more dramatically than other TM segments.

After having demonstrated the twisting motions of the whole receptor, the O ↔ C behavior of the pore channel (Figure 8C), and M2 bending based on the SRMD simulation results, we re-investigated changes in the ACh binding pockets. Because the clockwise motion is more plausible, we discuss the dynamics of the binding pockets based on the 1-ns SRMD simulation. As mentioned above, two ACh binding pockets, type-I and type-II, insert α^1^-γ and α^2^-δ interfaces, respectively. Conserved residues consisting of the two binding pockets were inspected during the 1-ns SRMD simulation. In agreement with the CMD simulation (Figure 2), the SRMD simulation also demonstrated the flexibility of the binding pockets. The SRMD profiles indicate that residues lining the binding pockets [44,45] experienced large movements (Figure 9). Similar to the CMD findings, SRMD results also indicate that C-loops are more flexible than A-loops and B-loops (Figure 9A and 9B). Unlike the results of CMD simulation (Figure 2A), the two binding pockets fluctuate along the SRMD trajectory, as shown in Figure 9C.

## Discussion

In this study we have elucidated the dynamic behavior of the nAChR using MD simulations, thus gaining insight into the gating mechanism of nAChR at the atomic level. Firstly, a 30-ns CMD simulation was performed on the cryo-EM structure of nAChR at 4 Å resolution [12]. Though this is much longer than any other CMD simulation carried out to date, it is still far from the physiological time scale of channel opening and closing transitions [46]. One of the major findings of this study is the collective motions of C-loops, M1, and M2 helices (Figure 2). Induced by the inward motion of the C-loop and the inward and upward motion of the outer β-sheet (β9 and β10) (Figure 2B), M1 and M2 undergo anticlockwise motions around the pore axis (Figures 2C and D). Although 30 ns is not a sufficiently long period, the CMD simulation was able to address the rotational motion of the entire receptor around the pore axis. The rotating speeds of the receptor domains are in the order MA domain > TM domain > EC domain (Figure 3), resulting in twisting motions of the EC domain to the TM domain and of the TM domain to the MA domain. In addition to rotation, M2 helices lining the pore channel undergo bending vibration (Figure 5A). Interestingly, M2 helices also exhibit a local twisting motion because the top part of the pore channel rotates faster than the bottom part (Figure 5B). Either the hinge of the bending vibration or the centre of twisting motion of M2 segments is located at the middle section of M2, near the L- and V-rings. This result suggests that the gate of nAChR is in the middle of M2, which is in agreement with recent results obtained using the single-molecule kinetic analysis method [26] and previous results using substituted cysteine or histidine accessibility methods [25]. In response to the twisting motion of the whole receptor in general and the synthetic motion of M2 helices in particular, the pore channel shrank during the 30-ns CMD simulation (Figure 4) and the L- and V-rings became significantly narrower (Figure 4A). This result implies that the cryo-EM structure of nAChR is not a fully closed state, and that a more closed state possibly exists, as predicted by our MD simulation (Figure 4B). However, due to the limitation of current MD methods, large protein assemblies such as nAChR cannot be simulated on a physiological timescale (∼millisecond). Our 30-ns CMD simulation did not detect the open state of nAChR. NMA not only detected the twisting motion of nAChR (Figures S2–S4), but also perceived the opening motion of the pore channel (Figure 6). Taken together, the CMD and NMA simulations suggest that the structural arrangement of nAChR intrinsically facilitates flickering of the receptor between the closed and open state, satisfying the gating reaction.

Recently, we have explored the causes of enforced rotation of AChBP [47,48] using the steered molecular dynamics (SMD) method, an approach often used for studying the dynamic and kinetic processes of ligand-receptor binding and unbinding [49–52]. Due to the high structural and sequence similarities between nAChR and AChBP, this SMD simulation may provide some clues as to the dynamics of the EC domain nAChR in response to ligand binding. The simulation revealed that the entire AChBP underwent a rotation concurrent with the ligand unbinding process [48]. This result implies that the reverse process, i.e., ligand binding, may drive AChBP to rotate in an opposite direction. The 30-ns CMD simulation revealed that nAChR has a tendency to rotate automatically around its pore axis. Binding of two ACh molecules to the receptor may accelerate the rotation process. To mimic the pulsive action of ACh binding, SRMD simulations were performed by assigning an angular momentum to the EC domain. The SRMD results revealed that clockwise rotation may produce conformational changes in the receptor which enable it to fulfill its gating functions. The orientation of the binding pockets also determined that the binding force of ACh can only drive the receptor to undergo a clockwise rotation. Indeed, the rotation of the EC domain induced a rotation of the TM domain, the former rotating faster than the latter (Figure 7), indicating that the SRMD also detected a twisting motion between the EC and TM domains. Remarkably, the SRMD simulation revealed an open–close (O ↔ C) change of the pore channel (Figure 8A and 8C). The displacement of ∼3.0 Å in pore channel diameter, measured in the vicinity of the L- and V- rings, is significant for the function of the receptor, because it has been suggested that even relatively minor displacements in the gate area can trigger the functional transition of the pore from closed to open [53,54]. This mechanical behavior of nAChR is in line with the experimental phenomenon: with the ligand bound, the channel still flickers between open and closed states, albeit now has a 90% probability of being in the open state [11].

Based on our MD simulation results and analyses, we are able to provide a clear correlation between the structural dynamics of nAChR and the gating mechanism induced by ligand binding. The binding of two ACh molecules to the receptor plays the role of handspikes, driving the EC domain to rotate around the pore axis like a turntable. The rotation of the EC domain propagates first to the M1 helices and then to the M2 helices, and the global rotations of M1 and M2 drive M3 and M4 to rotate. The rotation of the TM domain and the intrinsic bending vibration of M2 synthesize a twisting motion of the pore channel, the centre of the twist being in the middle of M2, where the gate is possibly located as recently detected using the single-molecule kinetic analysis method [26]. Tightening and relaxing of the twisting motion triggers the channel to flicker between open and closed states, which may be this receptor's ingenious “gating speciality”.

## Materials and Methods

### Simulation system.

To mimic the nAChR in a lipid bilayer environment, an initial 130 × 130 × 185 Å^3^ simulation box was built with the MD simulation package. The recent cryo-EM structure of the nAChR (PDB entry 2BG9) [12] was used as a starting structure. A large DPPC lipid bilayer [18,27], containing 405 lipid molecules, was constructed to generate a suitable membrane slab. The cubic box containing the nAChR/DPPC system was then filled with SPC water molecules [28]. Subsequently 205 Na^+^ and 140 Cl^−^ ions were added to neutralize the charge of the whole system. The entire simulation system consisted of 253,212 atoms.

### CMD simulation.

After energy minimization of the whole system, a total of 950 ps simulations were run, gradually releasing the force constraints on the lipid, hydrogen atoms, side-chain, main-chain except C_α_ atoms, and C_α_ atoms of the receptor. A 30-ns CMD simulation was then performed. All the simulations were carried out with a GROMACS package [55–57], using NPT and periodic boundary conditions. A modified GROMOS87 fore field [58] was applied for the protein and the lipid parameters adopted were those used in previous MD studies of lipid bilayers [18,59–62]. The particle-mesh Ewald algorithm [63,64] was adopted for the calculation of electrostatic interactions and the LINCS method [65] for constraining bond lengths. The integration step is 2 fs. To maintain protein, lipids, Na^+^ and Cl^−^ ions, and water molecules at a constant temperature of 323 K, the Berendsen thermostat was applied using a coupling time of 0.1 ps. The Berendsen pressure coupling was employed with semi-isotropic scaling that is considered appropriate for the membrane simulation [66]. A constant pressure of 1 bar was applied independently in *X*, *Y*, and *Z* directions.

### Normal mode analysis.

Normal mode analysis is a classical technique for generating functionally relevant movements of biological macromolecules [67]. We applied the elastic network model (ENM) [68–71] in which the structure is described as a set of pseudoatoms linked by harmonic springs if (and only if) their mutual distance is less than a given threshold (http://lorentz.immstr.pasteur.fr) [72,73]. We employed a cutoff of 10 Å as the default value to calculate the lowest-frequency modes. The twist-to-open motion was only observed in the lowest frequency mode.

### Rotational angle calculation.

In this study, a rigorous mathematical method was used to calculate the rotation of a body (e.g., EC domain or five M2 helices). For a body consisting of *n* atoms, its moment of inertia (*I*) can be expressed by

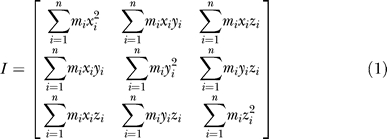
where *m_i_* is the mass of *i*th atom, (*x_i_*, *y_i_*, *z_i_*) is the coordinate of atom *i*. We can obtain the eigenvectors of Equation 1 by solving the eigenvalue problem. We define the three eigenvectors as the instant axes of the body, *X*-, *Y*-, *Z*-axis. At a given instant along the MD trajectory, the atoms of the body move to new positions, a new moment of inertia (*I′*) is obtained expressed by:

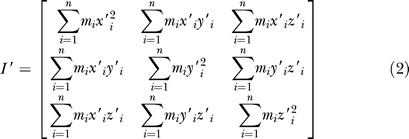
where *m_i_* is the mass of *i*th atom, 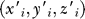 is the new coordinate of atom *i*. Similarly, we may get three new instant axes of the body (*X′*, *Y′*, *Z′*). Because of the symmetry of the receptor (with a 5-fold pore axis), the *Z′*-axis is always parallel to the pore axis of the receptor, so the rotation angle can be simply defined as the included angle between *X* and *X′* or between *Y* and *Y′*. In this way, all the rotation angles of the EC domain, TM (M1–M4) helices, and MA helices were calculated.

### SRMD simulations.

To simulate the rotation of nAChR around the pore axis, a nonequilibrium MD method was developed on the basis of the CMD method. The idea of this new MD method was to force the receptor to rotate around the pore axis by adding an angular momentum (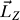 ) around the same axis. The angular momentum, 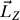 , can be calculated by Equation 3,

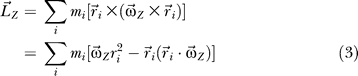



Giving an initial angular velocity 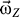 , the receptor will rotate around the *Z*-axis (also the pore axis). However, because of the friction between the receptor with water as well as with the lipid bilayer, the rotation will slow down. To keep the rotation of receptor, the angular momentum (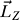 ) should be kept in conservation. To this end, the angular velocity (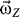 ) should be adjusted frequently. The instant angular velocity (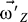 ) at any time of simulation is determined by Equation 4,

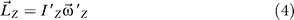



The moment of inertia (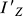 ) can be obtained by solving the eigenvalue problem of Equation 2, i.e., the eigenvalue corresponding to *Z′*-axis of Equation 2 is the value of 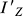 .

For practical purposes, the initial angular velocity (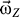 ) can be set at any value, depending on the simulation requirement. In this study, we set the magnitude of 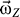 at 5 × 10^−4^ rad·ps^−1^ to allow the receptor to finish one cycle within ∼10 ns. Because the angular momentum was assigned to the EC domain, the instant moment of inertia (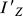 ) was calculated over all the atoms of EC domain.

All MD simulations were run on the DAWNING 4000A at Shanghai Supercomputer Center, having 2,128 AMD Opteron CPUs. Preparation, analysis, and visualization were performed on a 128-CPU Silicon Graphics Origin3800 server. Pore radius profiles were calculated using the program HOLE [36–38], and binding pocket volume was calculated using the program SURFNET [31].

## Supporting Information

Figure S1 The Time-Dependent RMSDs for the C_α_ Atoms of Each Subunit and Helix of the EC Domain (A) and Five M2 Helices (B) with Respect to Their Starting Structures (1.2 MB TIF)

Figure S2 Motions of nAChR Revealed by the First Mode of the Normal Mode Analysis (NMA) on the Snapshot Extracted from the 20th ns of the CMD Simulation Top (A) and side (B) views of periodic snapshots of the EC domain, as well as the corresponding top (C) and side (D) views of periodic structures of the M2 helices, are shown by superposing the starting conformation (green), and conformations at the one-fourth (red) and the three-fourth (blue) harmonic period.
(8.8 MB TIF)

Figure S3 Angular Displacements of the EC Domain, M1–M4 Helices, and MA Helices with Respect to the Starting Structure (280 KB TIF)

Figure S4 Angular Displacements of the Pore-Facing Rings in the M2 Helices Relative to the EC Domain with Respect to Their Starting Structures (413 KB TIF)

### Accession Numbers

The Protein Data Bank (http://www.rcsb.org/pdb/home/home.do) accession number of the nAChR used in this paper is 2BG9.

## Abbreviations:

AChBP: acetylcholine binding protein
CMD: conventional molecular dynamics
COM: center of mass
cryo-EM: cryo-electron microscopy
DPPC: dipalmitoylphosphatidylcholine
EC: extracellular
LBD: ligand-binding domain
LGIC: ligand-gated ion channel
MD: molecular dynamics
nAChR: nicotinic acetylcholine receptor
NMA: normal mode analysis
SPC: simple point charge
SRMD: steered rotation molecular dynamics
TM: transmembrane
